# A novel deep learning model for objective quantification of generalized anxiety disorder severity using EEG functional connectivity

**DOI:** 10.3389/fpsyt.2026.1764932

**Published:** 2026-02-18

**Authors:** Xiaodong Luo, Yuhuan Cui, Zihao Yan, Wei Liu, Bin Zhou, Gang Li, Shouqing Liu

**Affiliations:** 1 The Second Hospital of Jinhua, Jinhua, China; 2 College of Mathematical Medicine, Zhejiang Normal University, Jinhua, China

**Keywords:** beta rhythm, deep learning, electroencephalography, functional connectivity, gated multilayer perceptron, generalized anxiety disorder, severity assessment

## Abstract

Generalized anxiety disorder (GAD) is a prevalent and disabling psychiatric condition, yet its severity is still assessed mainly through clinical interviews and self-report scales, which lack objective neurobiological markers. This study aimed to develop an electroencephalography (EEG)-based deep learning (DL) model for objective quantification of GAD severity based on functional connectivity (FC) features. Resting-state EEG was recorded for 10 min from 80 patients with GAD and 39 healthy controls (HC). EEG segments with window lengths between 2 and 10 s were used to compute band-limited FC features, which were then used as input to a convolutional gated multilayer perceptron (Conv_gMLP) network for continuous prediction of the Hamilton Anxiety Rating Scale (HAM-A) total scores. The Conv_gMLP model achieved a mean absolute error (MAE) of 0.32 ± 0.07 in predicting the HAM-A total score (range: 0–56), outperforming conventional machine learning (ML) models and other DL architectures. Feature attribution analyses indicated that connectivity between frontal and temporal regions, particularly in the beta frequency range, contributed most strongly to the prediction of GAD severity. These findings suggest that EEG FC and beta rhythms encode clinically meaningful information about GAD severity, and that Conv_gMLP-based models may provide a promising tool for objective, time-efficient assessment to support individualized treatment planning.

## 1 Introduction

GAD is a chronic and recurrent mental illness characterized by excessive and uncontrollable worry across multiple aspects of life (1). It is frequently accompanied by symptoms such as irritability, difficulty concentrating, muscle tension, headaches, restlessness, and sleep disturbances, which significantly impair daily functioning and quality of life (2). In today’s fast-paced society, GAD has become one of the most prevalent psychiatric disorders (3), with recent studies reporting a prevalence rate as high as 6.0% (4). GAD not only disrupts social functioning and reduces overall well-being but also imposes a substantial economic burden on healthcare systems (5, 6). Notably, the severity of GAD is closely associated with the extent of social dysfunction (7), and patients with varying severity levels require tailored treatment strategies. Personalized treatment plans based on GAD severity can facilitate precision medicine, optimizing therapeutic outcomes (8). Therefore, accurately identifying and assessing the severity of GAD in a timely manner is crucial for effective patient management.

Although extensive research on GAD has been conducted over the years, an objective and quantitative assessment method for effectively evaluating the severity of GAD remains lacking in clinical practice. Currently, the evaluation of GAD severity primarily relies on clinical assessments and patient self-reports, both of which are inherently subjective (9). These evaluations are susceptible to individual differences and sociocultural influences, potentially compromising their reliability and consistency (10). The Hamilton Anxiety Rating Scale (HAM-A) is a widely used clinical instrument for assessing anxiety severity (11), and its total score served as the reference standard for severity labeling in this study (12). While the HAM-A is widely used in clinical settings to categorize anxiety severity into mild, moderate, or severe (13), it also presents several limitations. Its symptom descriptions often lack specificity, and the scoring process may be imprecise, further affecting the objectivity of severity assessment. Given these shortcomings, there is an urgent need to explore novel auxiliary diagnostic methods to enhance the accuracy and standardization of GAD severity assessment.

Although a variety of techniques have been adopted in current clinical research to assist in the auxiliary diagnosis and assessment of GAD—including EEG (14), functional magnetic resonance imaging (15), Facial Action Coding System (16), heart rate variability (17, 18)—studies have suggested that EEG may offer distinct advantages in evaluating the severity of the disorder (19). Owing to its non-invasive nature, low cost, and high temporal resolution, EEG remains a preferred modality for investigating human brain electrophysiology and cognitive function (20). EEG captures functional differences between brain regions by recording the dynamic electrical activity on the cortical surface or scalp. Neural activity can be reflected across multiple functional regions (21), and synchronous activity between different brain regions is referred to as functional connectivity (FC). Quantifying FC requires the application of specific computational estimators, and a variety of EEG-based FC estimators have been developed and widely utilized in the field of neuropsychiatry. For instance, Aydın et al. (22) demonstrated reduced segregation in resting-state EEG functional networks in patients with Alzheimer’s disease through EEG-based network analysis. Another study employed spectral coherence combined with ML to classify emotion regulation strategies (23). Among various estimators, Phase Lag Index (PLI) quantifies phase synchronization by assessing the asymmetry in the distribution of non-zero phase lags between signals, offering distinct advantages in estimating genuine brain connectivity. By focusing specifically on phase relationships between signals, PLI demonstrates enhanced stability and reliability in quantifying synchrony across different brain regions. Therefore, this work employs resting-state FC networks constructed using PLI, with the aim of objectively and continuously quantifying symptom severity in patients with GAD.

Currently, EEG-based research on GAD primarily focuses on classification tasks, typically involving binary classification (distinguishing healthy individuals from GAD patients) or multiclass classification (identifying mild, moderate, and severe GAD) (24, 25). However, such approaches largely remain at the level of categorical label prediction, falling short of meeting the clinical need for continuous, refined, and individualized assessment of GAD severity. Existing studies have shown that EEG combined with ML technology has great potential in the objective assessment of anxiety disorders (26–31), especially demonstrating excellent performance in the extraction and interpretation of high-dimensional EEG features (32). In comparison, DL algorithms, with their multi-layered neural network architectures, are capable of automatically extracting complex and abstract features from raw EEG signals, demonstrating superior performance in complex pattern recognition tasks (33). DL’s end-to-end learning capability, which integrates feature extraction and classification, has yielded remarkable results in handling high-dimensional, complex physiological data (34). In the broader field of EEG analysis, spatio-temporal representation learning has shown significant promise. For instance, in EEG-based emotion recognition, the STRFLNet model effectively captures both spatial and temporal features through representation fusion learning, demonstrating the importance of integrating multi-dimensional information for accurate brain state assessment (35). Meanwhile, some researchers have applied DL to EEG connectivity to assess the severity of social anxiety disorder, achieving objective prediction of anxiety severity (33). Although previous studies have indicated that DL models can assist in the diagnosis of GAD (36), reports on their use in precise and continuous severity assessment remain limited. Given this background, the integration of EEG techniques with advanced DL architectures holds promise for building high-accuracy predictive frameworks, enabling more efficient evaluation of GAD severity and further advancing its clinical application in precision diagnostics.

Building upon these considerations, a critical gap persists in the EEG-based deep-learning literature on GAD: the scarcity of frameworks that quantify symptom severity on a continuous, individual-level scale. To address this, the present study develops an EEG-based regression framework. This framework utilizes PLI-derived FC features and a novel Convolutional Gated Multilayer Perceptron (Conv_gMLP) architecture to predict the HAM-A total score. The central hypothesis is that resting-state FC patterns encode signatures relevant to clinical severity. This hypothesis is subsequently tested through systematic ablation studies of the Conv_gMLP architecture and an analysis of the impact of temporal resolution (time-window length) on predictive performance. Collectively, this work aims to bridge conventional symptom-based ratings with objective, neurophysiology-derived representations of GAD severity.

## 2 Materials and methods

### 2.1 Participants

The EEG data for this study were obtained from a local hospital. The GAD group consisted of 80 patients diagnosed with GAD, and the HC group included 39 HC. Patients with GAD were diagnosed by psychiatric experts using structured clinical interviews according to the Diagnostic and Statistical Manual of Mental Disorders, Fifth Edition (DSM-5) criteria, and anxiety severity was assessed using the HAM-A. The HAM-A consists of 14 items, each scored on a 0–4 ordinal scale, yielding a total score ranging from 0 to 56; higher scores indicate greater anxiety severity. In this study, the HAM-A total score was used as the reference standard for model training and evaluation. HC scores were retained as observed labels and included to represent the lower end of the continuous severity spectrum, without additional near-zero remapping. Detailed demographic information is presented in **Table 1**. As shown, there were no significant differences in age or sex between the two groups (age: *p* = 0.06; sex: *p* = 0.87), whereas the HAM-A scores showed a significant difference between the two groups (*p* = 1.14 × 10^−24^). To ensure data accuracy and reliability, all participants were required to meet specific inclusion criteria. First, all participants with GAD were first-time clinic visitors and had not taken any medications at the time of EEG acquisition. Furthermore, any participant with a current of using psychoactive medications (including but not limited to benzodiazepines, antidepressants, antipsychotics, or stimulants) was excluded. Second, all participants were right-handed, and individuals with epilepsy, neurodegenerative diseases, stroke, schizophrenia, or other psychiatric disorders were excluded. Additionally, participants with severe cardiopulmonary dysfunction, significant hepatic or renal impairment, malignant tumors, or autoimmune diseases were not eligible. These conditions and their treatments may influence cerebral oxygenation, metabolic status, inflammatory status, and neurophysiological activity, thereby potentially altering EEG rhythms and FC. To minimize potential confounding factors, participants were instructed to maintain adequate sleep the night before data collection and to refrain from smoking, as well as from consuming coffee or strong tea, within 8 hours prior to testing. Furthermore, individuals with a history of substance or alcohol abuse were excluded from the study. Lastly, participants were required to have no prior history of brain injury. This study was approved by the Ethics Committee of Zhejiang Normal University. Written informed consent was obtained from all participants prior to their involvement in the study.

**Table 1:** Demographic and clinical characteristics of participants.

Characteristic	HC (n=39)	GAD (n=80)
Age (years)	21-63 (37.3 ± 12.62)	22-74 (48.8 ± 11.2)
Sex (Male/Female)	12/27	21/59
HAM-A Score	2.3 ± 0.9	24.6 ± 8.1

### 2.2 EEG data acquisition and preprocessing

All EEG recordings were conducted in a specialized EEG acquisition room within the hospital to ensure a high-quality and standardized data collection process. Participants were instructed to stay awake, keep their eyes closed, and remain relaxed during each session, which lasted 10 min to record resting-state EEG signals. EEG data were acquired using a Nicolet EEG TS215605 system, with electrodes placed according to the international 10–20 system. A total of 16 electrodes were selected, specifically FP1, FP2, F3, F4, C3, C4, P3, P4, O1, O2, F7, F8, T3, T4, T5, and T6, with bilateral mastoids serving as reference electrodes. To ensure signal precision, the sampling rate was set to 250 Hz, allowing for the capture of subtle variations in EEG signals. Furthermore, electrode impedance was maintained below 5 kΩ to minimize signal interference and enhance data reliability.

The acquired EEG data underwent a series of preprocessing steps to ensure data quality and reliability: (1) Downsampling and Filtering: The raw EEG data were downsampled to 125 Hz and processed using a fourth-order Butterworth bandpass filter, restricting the frequency range to 4–30 Hz to remove unwanted low- and high-frequency noise. (2) Fast independent component analysis was employed to effectively remove artifacts, including eye blinks, electrocardiographic interference, and electromyographic noise, ensuring the extraction of clean EEG signals reflective of genuine neural activity. (3) Segmentation: The continuous EEG recordings were segmented into shorter epochs to capture distinct neural activity patterns and facilitate subsequent feature extraction. (4) Frequency Band Extraction: EEG signals were decomposed into distinct frequency bands to analyze different neural oscillations, including theta (4–8 Hz), alpha1 (8–10 Hz), alpha2 (10–13 Hz), and beta (13–30 Hz) rhythms. These preprocessing steps ensured that the EEG data were clean, reliable, and suitable for further analysis and research.

### 2.3 Feature extraction

In this study, PLI was extracted as a key feature for the quantitative assessment of anxiety severity in GAD patients. Compared to other time-domain and nonlinear features, PLI was considered more reliable in terms of stability. Time-domain features may exhibit fluctuations across different time points, leading to inconsistencies. Nonlinear features are highly susceptible to signal noise, affecting their robustness. PLI has been widely recognized for its advantages in assessing FC in brain networks (37, 38). Unlike time-domain features that can fluctuate with time and nonlinear features that are sensitive to noise, PLI focuses on the phase relationship between signals, which is more stable and reliable for quantifying synchronization between different brain regions (39). The utilization of PLI features helps minimize instability-related noise, thus enabling a more accurate representation of EEG activity.

Given two preprocessed EEG signals, $x_{1}\left(t\right)$ and $x_{2}\left(t\right)$, the instantaneous phase information is first obtained using the Hilbert Transform, as shown in Equation 1:

(1) $$\text{Φ}\left(t\right)=\text{x}\left(\text{t}\right)+\text{j}\frac{1}{\pi }P.V.\int_{-\infty }^{\infty }\frac{\text{x}\left(\tau \right)}{t-\tau }d\tau$$

Here, j  represents the imaginary unit, and P.V.  denotes the Cauchy principal value. At each time point t, PLI can be used to assess the phase synchronization between ${\text{ Φ}}_{1}\left(t\right)\;$ and ${\text{ Φ}}_{2}\left(t\right)\;$ by computing the phase difference. The phase difference is calculated as shown in Equation 2:

(2) $$\text{ΔΦ}\left(\text{t}\right)=\text{arg}\left({\text{Φ}}_{1}\left(t\right)\right)-\arg\left(\Phi_{2}\left(t\right)\right)$$

Here, arg  denotes the phase angle of a complex number. Subsequently, the signum function of the phase difference is defined as shown in Equation 3:

(3) $$f\left(t\right)=\{\begin{array}{c} 1,\;\;\;if\;\text{ΔΦ}\left(\text{t}\right)\epsilon \left(-\text{π},\text{π}\right]\\ 0,\;\;otherwise\;\;\;\;\;\; \end{array}$$

Finally, the PLI is defined as shown in Equation 4:

(4) PLI=|〈f(t)〉|

Here, |·| denotes the mean operation, while  〈·〉 represents the absolute value. The PLI value ranges from 0 to 1, indicating the degree of phase synchronization between brain regions. A PLI value close to 0 suggests an absence of phase synchronization, whereas a value approaching 1 indicates strong phase synchronization.

In this study, four frequency bands were analyzed: theta (4–8 Hz), alpha1 (8–10 Hz), alpha2 (10–13 Hz), and beta (13–30 Hz). For each frequency band, PLI was calculated for all possible electrode pair combinations, yielding N × (N - 1)/2 features, where N = 16 in this study. Consequently, a total of  4×120=480 features were extracted across the four frequency bands.

### 2.4 Conv_gMLP

**Figure 1:**
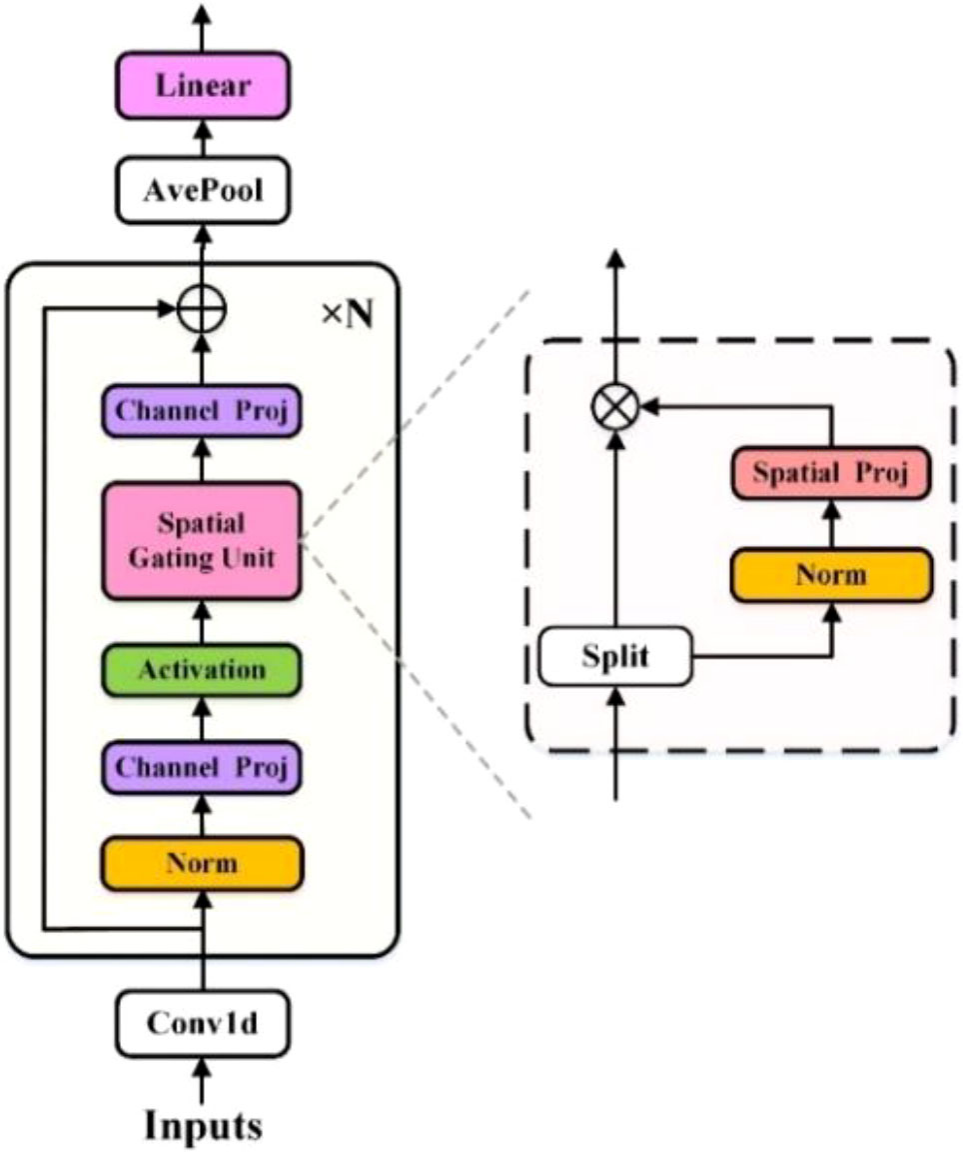
Schematic diagram of the Conv_gMLP model architecture with spatial gating units.

This section introduces the Conv_gMLP model, an innovative approach proposed for predicting the severity of GAD. The inspiration for Conv_gMLP originates from the gMLP module, which has demonstrated strong performance in image and natural language processing tasks (40). Given its frequent application in tabular data tasks, it is commonly referred to as tab_gMLP. In this study, the gMLP architecture is refined and extended to create the Conv_gMLP model. This novel gMLP model uses PLI features derived from quantitative EEG analysis as input. The Conv_gMLP model consists of three key components: channel expansion convolution module, the gMLP module, and feature aggregation. As illustrated in **Figure 1**, the Conv_gMLP Model architecture is constructed by stacking N identical gMLP blocks of the same structure and size. All projection operations within the model are linear, where ⊗ represents element-wise multiplication (linear gating) and ⊕ denotes element-wise addition (residual connection). Additionally, ablation experiments were conducted on different gMLP model architectures to evaluate the effectiveness of Conv_gMLP in predicting GAD severity.

#### 2.4.1 Channel expansion convolutional model

The traditional gMLP model is not suitable for processing inputs in the form of one-dimensional feature vectors. To address this, a 1D convolution (Conv1D) with a kernel size of 1 and a stride of 1 is applied to the 1×480 PLI feature input. This operation expands the number of channels, transforming and enhancing the features to enhance their representational capacity and diversity. This process is referred to as the channel expansion convolution module, with the specific formula shown in Equation 5:

(5) $$\tilde{x}=Conv1d\left(x,d_{channel},kernel\_size=1,\;stride=1\right)$$

Here, $d_{channel}$ represents the number of kernels, and $x\in {\mathbb{R}}^{1\times d_{feature}}$ is the feature vector input to the model, where $d_{feature}$ denotes the number of features. The output after channel expansion is denoted as $\tilde{x}\in {\mathbb{R}}^{d_{channel}\times d_{feature}}$.

The introduction of Conv1D is not merely a simple channel expansion operation; rather, it introduces parameterized spatial transformations in the feature dimension through convolutional operations. This operation can be viewed as a feature extraction process, which extracts more discriminative feature representations from the raw features, thus providing more valuable input for subsequent prediction tasks. With a kernel size and stride of 1, each convolutional kernel slides continuously over the feature dimension, focusing on individual feature points. Through local perception and abstraction, it captures subtle changes and patterns in the feature dimension. Furthermore, the use of multiple convolutional kernels allows each kernel to capture distinct abstract features along the feature dimension. This design enables the model to learn a broader range of feature representations at different levels, enhancing the model’s ability to express features in higher-dimensional spaces.

#### 2.4.2 gMLP module

The “g” in gMLP stands for gating, also known as the gated attention mechanism, which is a simple yet powerful attention mechanism that has been widely applied in neural network models in recent years. This mechanism dynamically adjusts the importance of the input, allowing the model to selectively focus on specific parts of the input, thereby enhancing the model’s performance and generalization ability. Liu et al. introduced a model called gMLP (40), which integrates gating into the Multi-Layer Perceptron (MLP) architecture. This model has demonstrated remarkable performance in key language and vision tasks.

The detailed pseudocode for a standard gMLP block is presented in **Table 2**. Each gMLP block further performs channel projection (CP) and statically parameterized spatial projection on the output data from the previous layer, aiming to enhance the representation and interaction of deep features. As shown in Equations 6–8, for simplicity, normalization, residual connections, and bias terms are omitted in the equations:

**Table 2:** Pseudo-code for the gMLP block.

***def*** *gmlp_block(x, d_channel, d_ffn):*
* shortcut = x*
* x = norm(x)*
* x = proj(x, d_ffn×2, axis=“channel”)*
* x = gelu(x)*
* x =* sp*atial_gating_unit(x)*
* x = proj(x, d_channel, axis=“channel”)*
***return*** *x + shortcut*
***def*** sp*atial_gating_unit(x):*
* u, v =* sp*lit(x, axis=“channel”)*
* v = norm(v)*
* v = proj(v, d_feature, axis=“spatial”, init_bias=1)*
***return*** *u×v*

(6) Z=σ(XU)

(7) $$\tilde{Z}=s\left(Z\right)$$

(8) $$Y=\tilde{Z}V$$

Here, σ represents the activation function, specifically the Gelu activation function used in this study. $X\in {\mathbb{R}}^{d_{feature}\times d_{channel}}$ denotes the output from the previous layer, while U and V define linear projections along the channel direction, with neuron counts of $2\times d_{ffn}$ and $d_{channel}$, respectively. s(·) refers to the Spatial Gating Unit (SGU), which performs spatial projection on high-dimensional features. SGU employs a simple spatial linear mapping and gating operation, which includes spatial dimensional operations, allowing it to capture interactions between spatial high-dimensional features and thereby enhance the representation of key features. SGU first splits the input data Z along the channel dimension into two parts ( $Z_{1}$ and $Z_{2}$), where $Z_{1}$ is linearly mapped to the spatial domain, and the output is then obtained by performing a dot product with $Z_{2}$, as shown in Equation 9:

(9) $$s\left(Z\right)=(Z_{1}W+b)⊗Z_{2}$$

Here, $W\in {\mathbb{R}}^{d_{feature}\times d_{feature}}$. To ensure training stability, W is initialized to near-zero values, and b is initialized to 1. This initialization ensures that each gMLP block behaves like a regular linear layer during the early stages of training, where each feature is processed independently, and the model gradually learns the interactions between features during the learning process.

#### 2.4.3 Feature aggregation module

For the output of the gMLP module, average pooling and a linear layer are used to reduce the dimensionality and integrate the high-dimensional features, mapping them to the final output space. Finally, the mean squared error loss function is employed to compute the error between the model’s output and the anxiety level indicator. The average pooling operation averages the features along the spatial dimension, generating a scalar value for each channel, thereby reducing the feature dimensionality. This helps capture the overall trend of the features and extract more representative ones. The computation of this module is given by Equation 10:

(10) out=AvePool(x, axis=“spatial”)×W+b

Among them, $W\in {\mathbb{R}}^{d_{channel}\times 1}$, AvePool represents average pooling, W is the weight, b is the bias.

### 2.5 Regression models

To compare the performance of the Conv_gMLP model, several ML and DL models were selected for this regression task. For all models, the n×480 PLI features were used as input. For n samples, each containing 480 PLI features, 80% of the data was used for training, and 20% was used for testing. The predictions were compared to the true values using the HAM-A score, and the MAE was calculated to assess the performance.

#### 2.5.1 ML models

Tree-based models, such as decision trees, have been widely used in disease detection and have shown excellent performance. In this study, LightGBM (Light Gradient Boosting Machine), XGBoost (eXtreme Gradient Boosting), and CatBoost (Categorical Boosting) were employed for the quantitative assessment of anxiety severity in patients.

1. CatBoost is an improved version of the Gradient Boosting Decision Tree (GBDT) algorithm, specifically designed for classification or regression tasks involving categorical features. It effectively addresses the imbalance and sparsity of categorical features by introducing an adaptive learning rate technique based on symmetric decision trees. In each iteration, the gradient boosting algorithm is used to construct decision trees, optimizing model performance by minimizing the loss function. Compared to traditional GBDT, CatBoost automatically handles the encoding of categorical features, eliminating the need for complex manual processing. Additionally, it offers automatic hyperparameter tuning, missing value handling, and a range of performance optimization strategies, such as random data sampling and feature importance evaluation.

2. XGBoost is a learning algorithm based on GBDT and is widely used in ML and data mining. It employs decision trees as base learners and constructs a powerful ensemble model by iteratively optimizing the gradient of the loss function. The underlying mechanism can be summarized as follows: during the construction of each tree, the gradient boosting algorithm is utilized to minimize the loss function, while a regularization term is introduced to control model complexity and prevent overfitting. Additionally, XGBoost incorporates a customized optimization strategy known as the “approximate greedy algorithm,” which efficiently leverages second-order gradient information of features to accelerate the training process. Ultimately, the final prediction output is obtained by aggregating the predictions of all weak learners.

3. LightGBM is an efficient ML algorithm based on GBDT, specifically optimized for large-scale datasets and high-dimensional features. It employs a “histogram-based decision tree” algorithm, which accelerates the training process by discretizing feature values and constructing histograms, thereby reducing memory consumption and computational complexity. In each iteration, the gradient boosting algorithm is used to construct decision trees, optimizing model performance by minimizing the loss function. Compared to traditional GBDT, LightGBM introduces a leaf-wise optimal split algorithm that precisely selects the best split points, further enhancing model accuracy. Additionally, it supports parallelized training and inference and provides a comprehensive set of hyperparameter tuning options and feature importance evaluation methods.

#### 2.5.2 DL models

Several DL models have also been employed for tabular data classification and compared with the performance of Conv_gMLP, including MLP and one-dimensional convolutional neural networks (1D CNN).

1. MLP is a feedforward artificial neural network model composed of multiple layers of neurons. Each neuron performs a nonlinear transformation of the input data using an activation function, with the Rectified Linear Unit (ReLU) function employed in this study. The network is trained through the backpropagation algorithm, iteratively updating parameters layer by layer to minimize the loss function. MLP exhibits strong fitting capabilities and nonlinear modeling potential, making it well-suited for capturing complex input-output relationships.

2. 1D CNN is a model based on Convolutional Neural Network (CNN), specifically designed for processing sequential data, such as time series or tabular data. It extracts features by applying one-dimensional convolution operations to the input data and reduces feature dimensionality through pooling operations. By stacking multiple convolution and pooling layers, the model progressively learns higher-level abstract features. Finally, classification is performed through fully connected layers and activation functions. The advantage of 1D CNN lies in its ability to effectively capture local patterns in data while maintaining parameter sharing and translation invariance. To enhance the model’s representational capacity, two linear modules, each consisting of a linear layer followed by a ReLU activation function, were added to the output layer.

### 2.6 Parameters optimization

ML and DL models contain numerous hyperparameters, and different combinations of these parameters yield varying training outcomes. Since hyperparameter selection significantly impacts model performance and generalization ability, optimizing these parameters helps mitigate the risks of overfitting or underfitting, thereby enhancing predictive accuracy and robustness. This study employ ed the Tree-structured Parzen Estimator (TPE) algorithm to search for the optimal hyperparameter configuration and improve model performance. Compared with traditional grid search or random search methods, TPE leverages historical data to accelerate the optimization process, offering lower time complexity and efficiently identifying superior hyperparameter sets in less time.

The TPE algorithm, implemented in the Hyperopt Python library, is a powerful method for hyperparameter optimization, comprising three key steps to ensure an effective and practical process. First, defining the objective function is fundamental to the TPE algorithm. The optimization target in this study is the MAE between the model’s predictions on the test set and the ground truth. Second, defining the hyperparameter search space is crucial. The hyperparameter search space used in this study is detailed in **Tables 3**–**8**, with the selection of ML optimization parameters and their respective ranges based on the AutoGluon automated ML framework. Finally, setting the number of search iterations controls the optimization process, with a maximum of 30 iterations specified. Through the systematic integration of these three steps, the TPE algorithm efficiently explores the hyperparameter space, enhancing both the performance and generalization capability of ML and DL models.

**Table 3:** LightGBM optimization variables and ranges.

Parameter	Description
num_leaves	uniformint[16,96]
min_data_in_leaf	uniformint[2,60]
feature_fraction	Uniform[0.75,1]
learning_rate	loguniform[log(5e-3),log(0.1)]

**Table 4:** XGBoost optimization variables and ranges.

Parameter	Description
Learning rate	loguniform[log(5e-3),log(0.1)]
depth	uniformint[3,10]
min_child_weight	uniformint[1,5]
colsample_bytree	uniform[0.5,1.0]

**Table 5:** Catboost optimization variables and ranges.

Parameter	Description
max_depth	uniformint[5,8]
l2_leaf_reg	uniform[1,5]
learning_rate	loguniform[log(5e-3),log(0.1)]

**Table 6:** MLP optimization variables and ranges.

Parameter	Description
num_layers	uniformint[1,2,3]
num_ffn	uniformint[128,256,384,512]
batch_size	uniformint[64,96,128,160]
learning_rate	loguniform[log(5e-5),log(1e-3)]

**Table 7:** Optimization variables and ranges of gMLP.

Parameter	Description
d_channel	uniformint[32,64,128,256]
num_layers	uniformint[1,2,4,6,8]
num_ffn	uniformint[128,256,384,512,640,768]
batch_size	uniformint [64,96,128,160]
learning_rate	loguniform[log(5e-5),log(1e-3)]

**Table 8:** Optimization variables and ranges of 1D CNN.

Parameter	Description
batch_size	uniformint [64,96,128,160]
learning_rate	loguniform[log(5e-5),log(1e-3)]

### 2.7 Evaluation metrics

In this study, repeated 5-fold cross-validation was employed to reduce estimation bias and improve the robustness of model evaluation. For ML models, four subsets of the data were used for training, while the remaining subset was used for testing to evaluate model performance. For DL models, 10% of the training data was further randomly selected for validation to prevent overfitting. All models underwent three rounds of repeated 5-fold cross-validation, and the final results were obtained by averaging the performance across all test sets. Model performance was assessed using the MAE, where a lower MAE indicates better model performance. The calculation formula is provided in Equation 11:

(11) $$MAE=\frac{1}{n}\sum_{i=1}^{n}\left|{\hat{y}}_{i}-y_{i}\right|$$

### 2.8 Grad-CAM visualization

Model interpretability has long been a critical research focus in the fields of artificial intelligence and ML. In this study, Gradient-weighted Regression Activation Mapping (Grad-RAM) is utilized to interpret the decision-making process of DL models. Grad-RAM builds upon Class Activation Mapping (CAM) and Gradient-weighted Class Activation Mapping (Grad-CAM), offering an advanced visualization technique.

CAM generates class-specific activation heatmaps by leveraging the feature maps from the last convolutional layer of a CNN with weights derived from the global average pooling layer, thereby revealing the key activation regions in the input signals. However, CAM struggles to capture detailed spatial information within feature maps. Grad-CAM addresses this limitation by incorporating gradient information to weight the heatmap, thereby improving the precision of interpretability and deepening insights into model decisions. This enables clear visualization of the regions critical to the model’s classification decisions. Consequently, Grad-CAM significantly enhances model interpretability, fostering a better understanding of DL model decision-making and increasing their reliability. Its mathematical formulation is detailed in Equations 12, 13:

(12) $$\alpha_{k}^{c}=\frac{1}{Z}\underset{i}{\sum }\underset{j}{\sum }\frac{\partial y^{c}}{\partial A_{ij}^{k}}$$

Here, $y^{c}$ represents the score predicted by the network for class c (before applying softmax activation), $A_{ij}^{k}$ denotes the data at position (ij) in channel k of the feature layer A, and Z refers to the width and height of the feature layer multiplied together.

(13) $$L_{Grad-CAM}^{c}=ReLU\left(\underset{k}{\sum }\alpha_{k}^{c}A^{k}\right)$$

Here, A represents a feature layer (the output of the last convolutional layer is taken in this study), k denotes the k-th channel in feature layer A, c represents the target class, $A^{k}$ refers to the data in the k-th channel of feature layer A, and $\alpha_{k}^{c}$ represents the weight of the k-th channel in feature layer A for class c.

Grad-RAM is an improved version of Grad-CAM designed for the interpretability of regression tasks. For regression models, the gradient is defined as the derivative of the inverse of the prediction bias, where a smaller bias leads to a larger gradient. Specifically, $L_{\text{Grad-RAM}}^{e}$ is derived from Equation 14, with the weight of each channel $\alpha_{k}^{e}$ calculated using Equation 15. The probability $y^{c}$ in Grad-CAM is replaced by $y^{e}$, which is computed from Equation 16, where ŷ is the predicted target value and y is the predicted value. $L_{\text{Grad-RAM}}^{e}$ changes according to the variation in the error bias.

(14) LGrad-RAMe=ReLU(∑kαkeAijk)

(15) $$\alpha_{k}^{e}=\frac{1}{Z}\underset{i}{\sum }\underset{j}{\sum }\frac{\partial y^{e}}{\partial A_{ij}^{k}}$$

(16) $$y^{e}=\frac{1}{\sqrt{{\left(\hat{y}-y\right)}^{2}}+1^{e-9}}$$

## 3 Results

**Figure 2:**
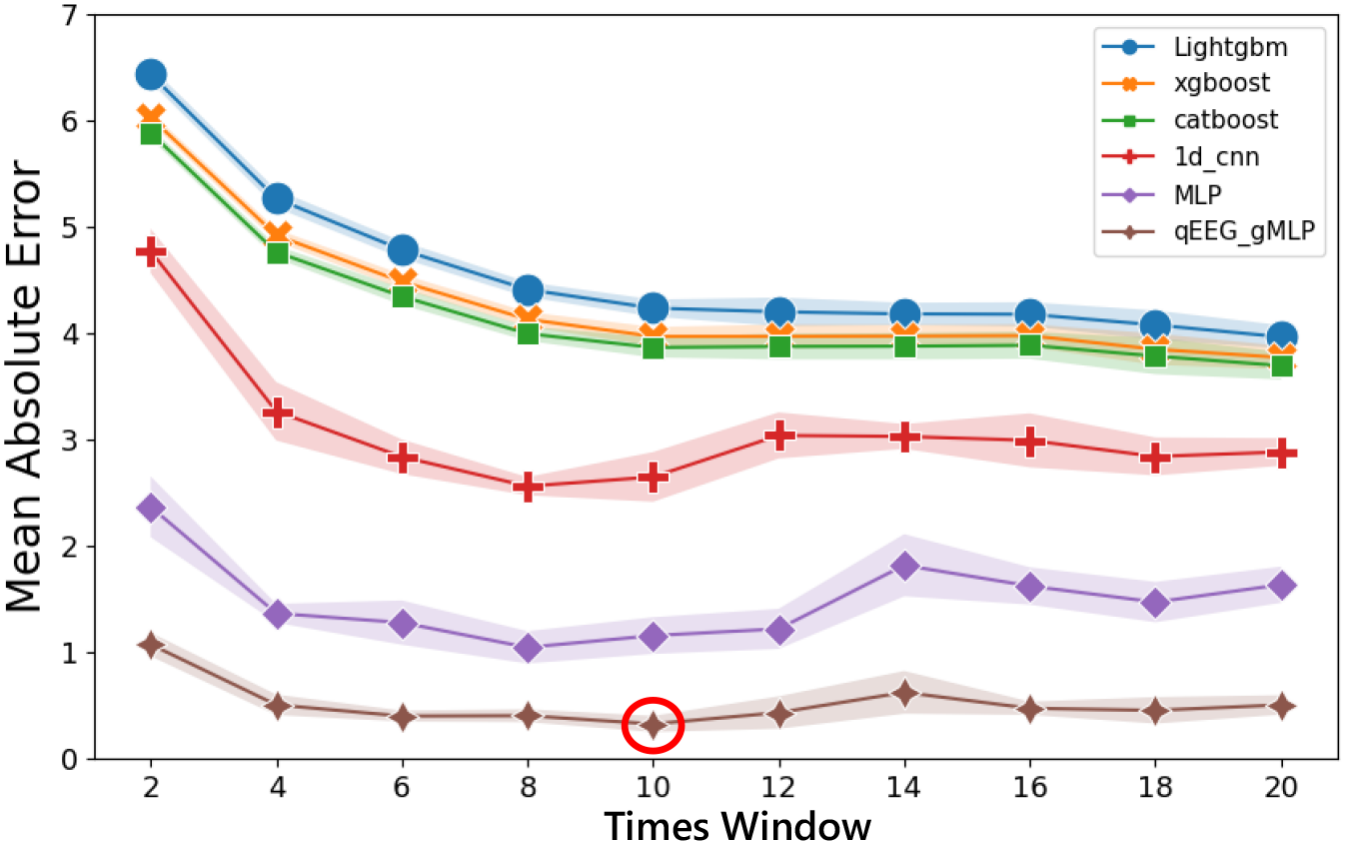
Regression performance of different time windows and different models.

This study explored the effect of different time window lengths on predictive performance, with the detailed results presented in **Figure 2**. The figure illustrates the regression performance of various models across different time windows. It was observed that for both traditional ML models (LightGBM, XGBoost, CatBoost) and DL models (1D CNN, MLP, Conv_gMLP), model performance gradually improved as the time window increased from 2 s to 10 s. However, beyond 10 s, DL models exhibited a decline in performance, whereas traditional ML models showed only marginal improvement. Notably, the proposed Conv_gMLP model achieved significantly lower prediction errors than all other models. The optimal predictive performance was obtained at a 10-second time window, yielding the lowest error of 0.32 ± 0.07, which was substantially superior to other models (LightGBM: 4.24 ± 0.08, XGBoost: 3.97 ± 0.09, CatBoost: 3.87 ± 0.09, 1D CNN: 2.65 ± 0.23, MLP: 1.16 ± 0.17).

Ablation experiments were conducted to explore the impact of different gMLP architectures on model performance. Conv_gMLP (SC) applies the SGU before the CP, allowing early spatial feature selection but potentially limiting subsequent channel interactions. In contrast, Conv_gMLP(CS) first employs CP to enhance feature representation across channels, followed by SGU to refine spatial dependencies, leading to more effective feature extraction. The experimental results are presented in **Table 9**. As shown in **Table 9**, each component of Conv_gMLP and its corresponding placement play a crucial role. In summary, the proposed Conv_gMLP model consistently outperformed other models across different time windows, demonstrating its significant advantage in evaluating the GAD dataset used in this study. This superiority stems from the model’s unique architectural design and the inherent strengths of the gMLP framework in handling nonlinear relationships and extracting complex features.

**Table 9:** Ablation experiment results.

Model	MAE
MLP	1.15 ± 0.12
Conv_gMLP (SC)	0.74 ± 0.14
Conv_gMLP (CS)	0.32 ± 0.07

**Figure 3:**
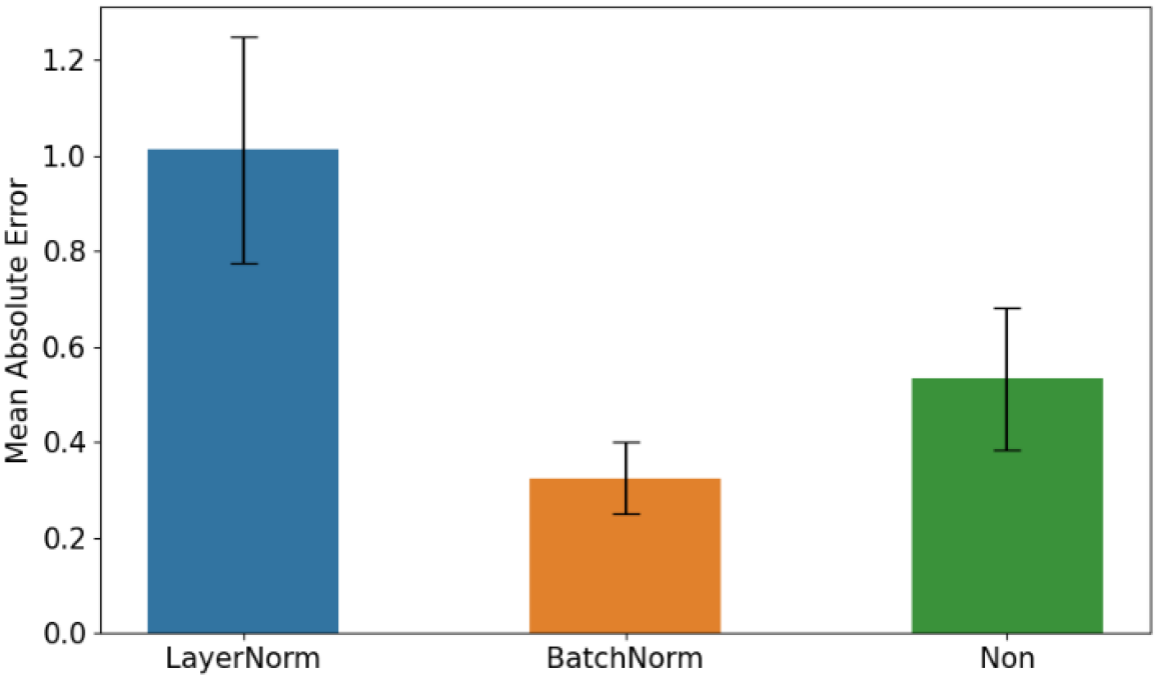
Different normalization methods in Conv_gMLP.

Furthermore, this study explored the effect of different normalization methods on predictive performance within a 10-second time window, comparing LayerNorm, BatchNorm, and no normalization (Non). Detailed results are presented in **Figure 3**. LayerNorm yielded the highest error at 1.01 ± 0.24, indicating suboptimal performance. In contrast, BatchNorm significantly reduced both error and standard deviation to 0.32 ± 0.07, while the error without any normalization was 0.53 ± 0.24. Based on these findings, BatchNorm was selected as the preferred normalization method due to its superior performance in minimizing error. Among the tested methods, LayerNorm exhibited the worst performance, yielding the highest error. This may be attributed to the fact that LayerNorm normalizes each sample independently at every layer of the neural network. For quantified EEG feature data, it considers only the statistical properties of individual samples while ignoring inter-sample statistics. The high complexity of the experimental data may have hindered LayerNorm’s ability to effectively normalize the features, ultimately impairing model performance. In comparison, BatchNorm demonstrated the best performance in this study, significantly reducing both the error and standard deviation. This improvement is likely due to BatchNorm’s ability to account for the statistical properties of mini-batches, allowing it to better capture the global characteristics of EEG feature data. Moreover, BatchNorm’s inherent regularization properties help mitigate overfitting, enhancing the model’s generalization capability.

**Figure 4:**
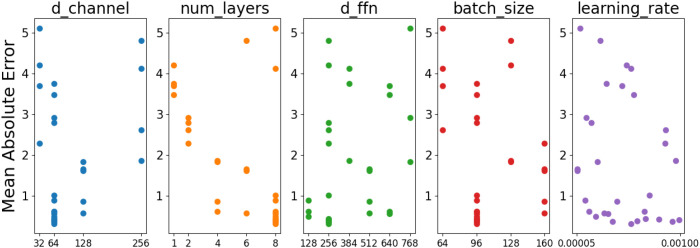
Performance of parameter combination optimized by TPE method.

Hyperparameter optimization of the 10-second Conv_gMLP model under BatchNorm settings was performed using the TPE optimization algorithm in this study. **Figure 4** illustrates the parameter search space. The results showed that the optimal fitting performance was achieved when the d-channel was set to 64, the num-layers was 8, the feedforward network dimension (d-ffn) was 256, the batch size was 96, and the learning rate was 0.00055, yielding a minimum MAE of 0.325. This finding indicated that under this specific combination of hyperparameters, the model effectively captured underlying data patterns and achieved lower prediction errors. Compared to other hyperparameter configurations, this combination optimally balanced model complexity and training stability, thereby preventing overfitting and enhancing generalization capability.

**Figure 5:**
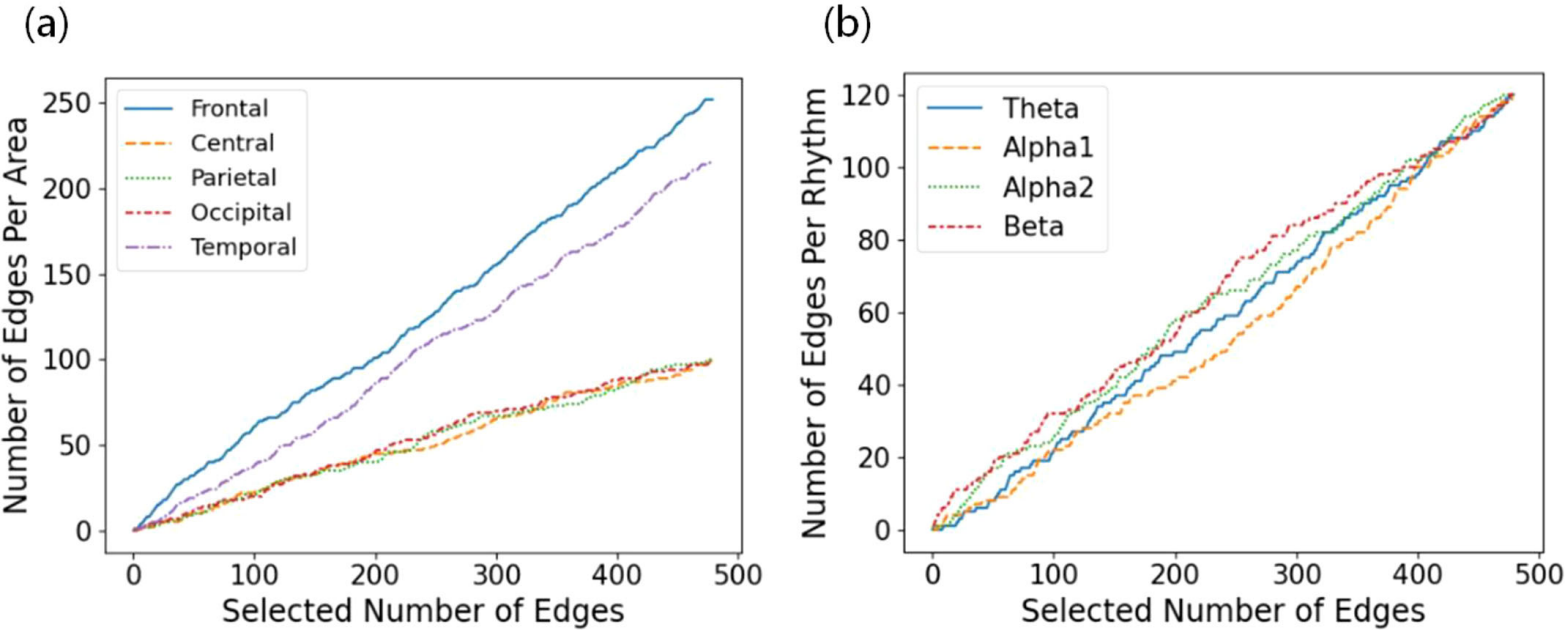
**(a)** Distribution of feature weights across different brain regions; **(b)** Distribution of importance across different EEG rhythm features.

Finally, the Grad-RAM method was utilized to compute the feature weights, which were subsequently ranked from highest to lowest. The results, presented in **Figure 5a**, illustrate the distribution of feature importance across different brain regions. The analysis revealed that the frontal lobe exhibited the highest feature weight among all brain regions, followed by the temporal lobe, underscoring the critical role of these regions in the brain functional activity of GAD patients. Moreover, **Figure 5b** depicts the distribution of rhythmic features across brain regions revealing that, although the boundaries of importance among various rhythmic features were somewhat ambiguous, the feature weight of the beta rhythm was notably higher. Further analysis indicated that GAD patients with different severity levels displayed distinct abnormalities in brain FC strength and the number of connections. These differences could serve as potential biomarkers for differentiating the severity of GAD. Thus, the EEG-based feature weight analysis provides a novel quantitative basis for the diagnosis of GAD and estimation of its severity.

## 4 Discussion

This study introduces an innovative approach to assessing GAD severity by applying the Conv_gMLP model to resting-state EEG signal processing. The main findings of the study are as follows: First, the Conv_gMLP model demonstrated significantly lower prediction MAE compared to other models, highlighting the effectiveness of its architectural design and feature extraction strategy. Second, FC analysis revealed that the frontal and temporal lobes played a crucial role in distinguishing GAD severity, with beta rhythms emerging as the most informative feature. These findings underscore the importance of both the model’s predictive capability and the neurophysiological insights derived from EEG-based analysis, which will be further elaborated in the following sections.

### 4.1 Time-window optimization and architectural innovation of Conv_gMLP: enhancing precision in GAD severity assessment

This study demonstrates that the Conv_gMLP model significantly outperforms other models in predicting the severity of GAD, owing to its innovative architectural design and optimized feature extraction strategy. Two key factors drive its superior performance: the optimization of the time window and the innovative structure of the Conv_gMLP model.

First, the selection of the optimal time window is critical for enhancing model performance. Previous studies have indicated that the selection of sliding time windows influences the quantification of brain network stability and detection performance, suggesting that optimizing this parameter can enhance predictive accuracy (41). Furthermore, it has been confirmed that a time window of approximately 10 s is most suitable for automatic EEG feature extraction and emotion state recognition, effectively capturing emotion-related oscillatory characteristics (42). This duration aligns with the principles of neurophysiology, as EEG signals can capture dynamic neural network activities. For instance, the beta rhythm oscillations, which are highly sensitive to anxiety levels (43), reflect the heightened brain alertness and arousal in GAD patients (44). Moreover, human emotional states, including anxiety, typically last for 10 s or longer (45). By systematically varying the sliding-window length, a 10-s epoch yielded optimal performance, with the ensemble learner attaining 98.1% accuracy for GAD-severity grading under this temporal window (24). Thus, the 10-second window effectively captures these oscillatory features, providing a biologically grounded explanation for the model’s superior performance.

Second, the Conv_gMLP architecture leverages neurobiological insights to improve GAD severity prediction. Anxiety disorders are associated with aberrant large-scale functional network patterns, characterized by enhanced connectivity between the insula and thalamus and reduced activity within the frontoparietal network—patterns that provide insight into the underlying pathophysiological changes in anxiety disorders (46). Notably, Chu et al. observed that GAD patients exhibit significantly stronger high-beta-band FC between brain regions than HC, and that this hyper-connectivity becomes more pronounced with longer illness duration (47). Functional networks are collections of brain regions with closely coordinated activity during both resting-state and cognitive tasks, each supporting distinct aspects of cognition. The Conv_gMLP model integrates quantitative EEG analysis with the gMLP framework (40), enabling precise identification of EEG activity patterns in GAD patients by extracting PLI features from EEG signals. This design reflects a deep understanding of both the data structure and the underlying neural mechanisms. In conclusion, the Conv_gMLP model successfully predicts the severity of GAD, owing to its optimized time window selection and innovative architectural design. These aspects not only enhance the model’s technical performance but also strengthen its alignment with the neurophysiological basis of anxiety. Future research should focus on refining this model and exploring its potential in clinical applications.

### 4.2 Key factors in GAD severity evaluation: brain regions, beta rhythms, and FC

This study investigates the neurophysiological underpinnings of GAD severity, emphasizing the roles of brain regions, beta rhythms, and FC in characterizing the condition. Persistent excessive worry in GAD is strongly associated with structural and functional brain alterations (48), and identifying these functional changes may uncover disruptions in neural circuits and emotional regulation. Using the Grad-RAM method for feature weight analysis, this study suggests that severity-relevant features are primarily concentrated in the frontal and temporal lobes. Mechanistically, the prominence of frontal and temporal regions may reflect the involvement of large-scale networks supporting cognitive control, affective processing, and their interaction, which are broadly relevant to anxiety symptom expression. Studies indicate that the frontal cortex plays a crucial role in emotional cognition (14, 49). FC analysis further confirms aberrant prefrontal network connectivity in anxiety disorders (46) and highlights abnormalities in the temporal region of patients with GAD (25). Further studies reveal that most brain regions in GAD patients exhibit elevated correlation dimension (D2) values, particularly in the left prefrontal and right temporal lobes (50). Because correlation dimension is a nonlinear measure reflecting the complexity of EEG dynamics and information processing, such elevations in these regions may indicate altered cortical dynamics associated with greater anxiety severity. This finding further reinforces that key brain signals for assessing GAD severity primarily originate from the prefrontal and temporal lobes, consistent with our results.

Moreover, EEG rhythms encode extensive neural activity information and have been widely applied in both research and clinical assessments of GAD (51, 52). The EEG features of beta rhythms are significantly correlated with brain activity levels, with increased beta-band activity closely associated with anxiety symptoms (52, 53). Multiple studies have demonstrated that beta rhythm power is significantly elevated in GAD patients (53–55), strongly associated with brain alertness and arousal states (56), and more easily detectable under anxiety conditions (57). Mechanistically, heightened beta power may reflect persistently increased arousal and reduced network flexibility, which could make beta-band FC particularly informative for continuous severity estimation. Beta rhythm EEG features have been quantitatively utilized in GAD assessment. Recent evidence demonstrates significantly elevated beta power together with aberrant long-range fronto-temporal FC in patients relative to HC (43). This convergence may reflect stronger long-range coupling that supports sustained arousal, which could be relevant to severity estimation. Our study identifies beta rhythms as the most critical among EEG rhythm features, further confirming their value in evaluating GAD severity and providing a plausible biological basis for the observed predictive contributions.

Finally, the study suggests that EEG-based FC may serve as a valuable biomarker for distinguishing individuals with GAD (47). FC reflects the coordination and interactions among different brain regions, with alterations observable even in unconscious states. Variations in FC indicate alterations in brain region coordination and cognitive function, capturing specific traits of GAD (58). These connectivity disruptions are especially pronounced in GAD, where frontal-temporal connectivity strength is strongly associated with cognitive function. Overall, this study confirms the impact of brain regions and beta rhythms on GAD severity. It also underscores the importance of FC in brain coordination and cognition, suggesting its potential as a biomarker for assessing GAD severity.

### 4.3 Limitations

Although this study offers new evidence supporting a novel approach to quantifying the severity of GAD, several limitations should be acknowledged. First, the sample size was relatively small, and the sex distribution was uneven. Therefore, the generalizability of the proposed EEG-based severity estimation framework across sexes should be interpreted cautiously. Future studies should aim to increase the number of participants and ensure balanced sample sizes across groups. Second, the HAM-A was used as the reference standard for quantifying GAD severity. However, because the HAM-A is a clinician-rated and ordinal measurement instrument, it is subject to measurement variability, which may partly manifest as label noise in the model training data. Accordingly, the model’s prediction error reflects not only the potential gap between EEG-derived neurophysiological features and symptom severity, but also the intrinsic uncertainty of the clinical reference standard itself. Conceptually, our model learned a mapping between EEG features and the contemporaneous clinical consensus on severity. Future work should integrate multimodal assessments and longitudinal outcomes to establish a more robust composite standard, thereby further improving the validity and clinical applicability of objective severity evaluation. Third, the cross-sectional design limits the ability to evaluate the model’s performance in longitudinal monitoring or in predicting treatment responses. Prospective studies with repeated EEG measurements over time are necessary to determine the model’s utility in tracking disease progression. Finally, the current model was trained using data from a single clinical center, which may introduce regional bias and restrict its generalizability. Future research should prioritize multicenter validation to ensure the model’s robustness across diverse populations and healthcare settings.

## 5 Conclusion

In conclusion, this study introduced a quantitative approach to assessing the severity of GAD and successfully applied the Conv_gMLP model for its objective evaluation, thereby further validating the feasibility of using algorithmic models for severity quantification. The Conv_gMLP model achieved the lowest MAE (0.32 ± 0.07) in predicting GAD severity within a 10-second time window, significantly outperforming traditional ML models as well as other DL models. Additionally, the FC analysis conducted in this study provided evidence of altered brain network interactions in GAD patients, particularly showing enhanced connectivity between the frontal and temporal lobes. Regarding rhythmic features, beta rhythm features were found to carry noticeably higher weights across brain regions. Based on these findings, the continued development and application of algorithmic models such as Conv_gMLP hold great promise for enabling more accurate and objective diagnosis and assessment of GAD severity, thereby supporting precision diagnosis, individualized treatment, and the advancement of clinical care for GAD.

## Data Availability

The datasets generated and analysed during the current study are not publicly available due to participant privacy and ethical restrictions, but are available from the corresponding authors on reasonable request.
